# Factors associated with initial treatment and survival for clinically localized prostate cancer: results from the CDC-NPCR Patterns of Care Study (PoC1)

**DOI:** 10.1186/1471-2407-10-152

**Published:** 2010-04-19

**Authors:** Maria J Schymura, Amy R Kahn, Robert R German, Mei-Chin Hsieh, Rosemary D Cress, Jack L Finch, John P Fulton, Tiefu Shen, Erik Stuckart

**Affiliations:** 1 New York State Cancer Registry, New York State Department of Health, 150 Broadway, Suite 361, Menands, NY 12204-2719, USA; 2 Division of Cancer Prevention and Control, Centers for Disease Control and Prevention, Atlanta, GA, USA; 3 Louisiana Tumor Registry, Louisiana State University Health Sciences Center, New Orleans, LA, USA; 4 California Cancer Registry, Public Health Institute, Sacramento, CA, USA; 5 Colorado Central Cancer Registry, Colorado Department of Public Health and Environment, Denver, CO, USA; 6 Rhode Island Cancer Registry, Rhode Island Department of Health, Providence, RI, USA; 7 Division of Epidemiologic Studies, Illinois Department of Public Health, Springfield, IL, USA; 8 South Carolina Central Cancer Registry, South Carolina Department of Health and Environmental Control, Columbia, SC, USA

## Abstract

**Background:**

Despite the large number of men diagnosed with localized prostate cancer, there is as yet no consensus concerning appropriate treatment. The purpose of this study was to describe the initial treatment patterns for localized prostate cancer in a population-based sample and to determine the clinical and patient characteristics associated with initial treatment and overall survival.

**Methods:**

The analysis included 3,300 patients from seven states, diagnosed with clinically localized prostate cancer in 1997. We examined the association of sociodemographic and clinical characteristics with four treatment options: radical prostatectomy, radiation therapy, hormone therapy, and watchful waiting. Diagnostic and treatment information was abstracted from medical records. Socioeconomic measures were derived from the 2000 Census based on the patient's residence at time of diagnosis. Vital status through December 31, 2002, was obtained from medical records and linkages to state vital statistics files and the National Death Index. Multiple logistic regression analysis and Cox proportional hazards models identified factors associated with initial treatment and overall survival, respectively.

**Results:**

Patients with clinically localized prostate cancer received the following treatments: radical prostatectomy (39.7%), radiation therapy (31.4%), hormone therapy (10.3%), or watchful waiting (18.6%). After multivariable adjustment, the following variables were associated with conservative treatment (hormone therapy or watchful waiting): older age, black race, being unmarried, having public insurance, having non-screen detected cancer, having normal digital rectal exam results, PSA values above 20, low Gleason score (2-4), comorbidity, and state of residence. Among patients receiving definitive treatment (radical prostatectomy or radiation therapy), older age, being unmarried, PSA values above 10, unknown Gleason score, state of residence, as well as black race in patients under 60 years of age, were associated with receipt of radiation therapy. Overall survival was related to younger age, being married, Gleason score under 8, radical prostatectomy, and state of residence. Comorbidity was only associated with risk of death within the first three years of diagnosis.

**Conclusions:**

In the absence of clear-cut evidence favoring one treatment modality over another, it is important to understand the factors that inform treatment selection. Since state of residence was a significant predictor of both treatment as well as overall survival, true regional differences probably exist in how physicians and patients select treatment options. Factors affecting treatment choice and treatment effectiveness need to be further explored in future population-based studies.

## Background

Despite the large number of men diagnosed with localized prostate cancer, there is as yet no consensus concerning appropriate treatment. In response to two Institute of Medicine reports suggesting that central cancer registries be utilized for tracking cancer treatment [1,2] the Centers for Disease Control and Prevention's (CDC) National Program of Cancer Registries (NPCR) undertook a multi-state Patterns of Care study to identify cancer treatment in a cross-section of the United States. In order to include follow-up data for survival analysis in the project, the study design was retrospective, based on cancers diagnosed in 1997.

According to the National Cancer Institute's (NCI's) Physician Data Query from 1996, acceptable treatment options for localized prostate cancer included surgical removal of all of the prostate and some surrounding tissue (radical prostatectomy); radiation therapy, either external beam or interstitial (brachytherapy); and careful observation without further immediate treatment ("watchful waiting"). Hormone therapy, another option, could be achieved by either orchiectomy (surgical removal of the testicles) or the administration of drugs to block hormone production or action [3]. These recommendations have not changed substantially in the intervening years [4]. According to the current Clinical Practice Guidelines of the National Comprehensive Cancer Network [5], observation, radiation, and radical prostatectomy are all reasonable treatment options for localized prostate cancer, the treatment decision being based on balancing expected long-term survival in the absence of treatment against individual tolerance for the side effects of current therapies.

### Objectives

The objectives of the current study were to describe the initial treatment patterns for localized prostate cancer in a population-based sample and to determine the clinical and patient characteristics associated with initial treatment. In particular, what factors are related to choice of conservative management versus definitive treatment; and among patients receiving definitive treatment, what factors are related to radiation therapy versus radical prostatectomy? Radical prostatectomy and radiation therapy are viewed as definitive treatment for localized prostate cancer; that is, they are potentially curative because they remove or destroy cancerous tissue. Hormone therapy and watchful waiting are considered non-curative and are classified as conservative management. A secondary objective of the current study was to determine whether mode of initial treatment for localized prostate cancer was related to patient survival.

## Methods

The CDC-NPCR Breast, Colon, and Prostate Cancer Data Quality and Patterns of Care Study (PoC1) involved seven participating states (California, Colorado, Illinois, Louisiana, New York, Rhode Island, and South Carolina) and the CDC. The sampling methodology for this study has been explained in detail elsewhere [6,7]. In brief, random samples of cancer patients diagnosed in 1997 were selected from the databases of the participating central cancer registries. Institutional Review Board approval for use of the data was obtained at every participating university or health department and at the Centers for Disease Control and Prevention.

### Inclusion Criteria

Patients included in the current analysis were those diagnosed in 1997 with a first primary prostate cancer (ICD-O-2: C61.9; Behavior = 3; sequence number 00 or 01), diagnosed at a clinically localized stage. Since the determination of pathologic staging requires histologic examination of the resected specimen, usually involving total prostatoseminalvesiculectomy and pelvic lymph node dissection [8], pathologic staging would only be available for patients who had undergone radical prostatectomy. Patients were considered to have clinically localized disease if (1) the tumor was clinically inapparent (i.e., not palpable or visible by imaging) or (2) the tumor was confined within the prostate (cT1c and cT2), and there was no clinical evidence of nodal involvement and no evidence of metastasis. Frequently, when pathologic staging information was available in the medical record, clinical staging information was not documented. Therefore, patients with pathologic assessment of the tumor (pT1 and pT2) were also included as long as there was no clinical evidence of nodal involvement and no evidence of metastasis. Additionally, only patients with microscopically confirmed adenocarcinomas who were alive six months post diagnosis were included in the analysis.

### Measurement

Hospital medical records of the study cases were reabstracted by specially trained staff in each state, using a common data entry form. Demographic, diagnostic, staging, and treatment data were obtained for each patient from hospital charts, supplemented by records from physicians' offices and from ambulatory surgery and radiation therapy facilities. Because the proportion of Hispanics residing in many parts of the study area was low, Hispanic ethnicity was frequently not noted in the medical record (e.g., the hospital intake form did not include an ethnicity check-off). Patients were categorized as non-Hispanic unless there was evidence to the contrary (e.g., reference to Hispanic ethnicity in the history and physical section of the medical record). Race and ethnicity information was combined to create four groups: Hispanic, non-Hispanic White, non-Hispanic Black, and non-Hispanic other (including Asian/Pacific Islander). ICD-9-CM codes that were recorded in medical records were abstracted to assess the presence of comorbid illness. Codes for the 17 conditions that make up the Charlson index were consolidated into a summary Charlson measure [9]. Insurance status was also determined from medical records. Private insurance included those with either private insurance or Medicare with a supplement. Public insurance included those with Medicare, Medicaid or welfare, or other federally funded health insurance. For a small proportion of patients, insurance type was indicated as "other".

Each patient's address was geocoded at the participating registry to the census tract level. The records were subsequently linked by census tract to the following socio-demographic indicators, derived from the 2000 census data: poverty (<20% versus 20%+ of residents below the 2000 poverty level); education (<25% versus 25%+ of residents age twenty-five and over with less than a high school education); working class status (<66% versus 66%+ working class occupations); and urban-rural residence (totally urban, totally rural, urban-rural mix, or unknown). Indicators were selected based on the work of Nancy Krieger [10]. Vital status as of December 31, 2002, was obtained from medical records, physician offices, and by linking to state vital statistics files and to the National Death Index (NDI) for 1997 through 2002. At the time funding for the study ended, 2002 was the last year for which data from NDI were available.

Initial treatment was defined as treatment received within the first six months following diagnosis. A hierarchical variable was created to categorize treatment, ranging from the most aggressive to the least aggressive [11]. Men who received radical prostatectomy were assigned to radical prostatectomy, whether or not they received any other therapy. Men who received radiation therapy (external beam or interstitial) were assigned to radiation therapy, whether or not they also received hormone therapy. The hormone therapy category was comprised of men who only received hormone therapy (medical or surgical). Men who had no record of any therapy within the first six months following diagnosis were assigned to the watchful waiting category. Whether or not cases with missing treatment data were excluded from analysis depended on what treatment data were missing relative to the treatment hierarchy. For example, patients with missing surgery information were excluded. However, patients with missing hormone information were still eligible for inclusion in the radical prostatectomy or radiation therapy categories. Patients who died within six months of diagnosis were deleted from all analyses because determination of treatment mode was based on the six month post-diagnosis timeframe.

### Data Analyses

We examined the proportion of patients receiving each type of therapy by sociodemographic and clinical factors. Bivariate analysis was used to examine the association between the four major treatment options and patient and tumor characteristics. The analytic variables included age at diagnosis, race/ethnicity, marital status, insurance, education, poverty, working class status, urban-rural residence, registry, whether the tumor was screen detected, digital rectal exam (DRE) results, PSA value, Gleason score, and Charlson comorbidity score. Two-sided chi-square tests were used to compare the distribution of treatments across the predictor variables. Variables found to be significantly associated in the bivariate analysis were included in the multivariable logistic regression models. For the multivariable analyses, marital status was dichotomized into married and not married (single, separated, divorced, and widowed). "Other" insurance was combined with private insurance. Separate logistic regression analyses were conducted to identify factors associated with receipt of conservative management and factors associated with receipt of radiation therapy among those receiving definitive treatment. A backward selection approach was utilized. That is, non-significant and non-relevant variables were deleted from the model one at a time. At each step, the change in parameter estimates for the remaining variables was examined to determine whether non-significant variables should be retained to control for confounding. The Hosmer-Lemeshow test was used to assess the goodness of fit of the model.

We examined all cause survival from diagnosis through December 31, 2002, conditional upon having survived six months following diagnosis (i.e., patients had to have survived six months to be included in this study). The proportion of patients surviving five years following diagnosis was estimated using the life table method. Cox proportional hazards models were used to estimate crude and adjusted hazard ratios. A backward selection approach was utilized to determine which factors to include in the final model. Time-varying covariates were used to test the proportional hazards assumption, which was violated for comorbidity. Therefore, time-varying covariates for comorbidity were retained in the final model. SAS statistical software (version 9.1, SAS Institute, Cary, NC) was used for all analyses.

## Results

A total of 3,504 patients with localized primary prostate cancer were identified. Of these, 176 were excluded from analysis for the following reasons: diagnosis year was not 1997 (n = 3), tumor was not microscopically confirmed (n = 23), histology other than adenocarcinoma (n = 3), month of diagnosis was unknown (n = 51), information on treatment or treatment dates was missing (n = 64), or died within six months of diagnosis (n = 53). Some patients were excluded for multiple reasons. A total of 3,328 patients with localized prostate cancer were left for the analysis of conservative versus definitive treatment. Twenty-eight of these patients did not have a radical prostatectomy or receive radiation therapy but were missing information on hormone therapy. Consequently, they could be included in conservative management but not in the specific treatment groupings shown in Table 1.

**Table 1:** Distribution of initial treatment by sociodemographic and economic characteristics of the patient.

							Percent receiving each therapy				
						
Characteristic			**Number**^**1**^	(%)	**Number**^**2**^	(%)	Radical prostatectomy	Radiation therapy	Hormonal therapy	Watchful waiting	**P**^**3**^
Total			3,328	(100)	3,300	(100)	39.70	31.42	10.27	18.61	
									
Age at diagnosis											
<60			602	(18)	601	(18)	72.88	15.14	3.00	8.99	<0.0001
60-64			568	(17)	565	(17)	61.42	21.06	5.13	12.39	
65-69			736	(22)	730	(22)	48.36	31.92	7.26	12.47	
70-74			709	(21)	703	(21)	20.91	47.08	10.81	21.19	
75-79			456	(14)	453	(14)	4.42	49.23	17.88	28.48	
>= 80			257	(8)	248	(8)	2.02	16.13	33.06	48.79	
Race/ethnicity											
Non-Hispanic White			2,671	(80)	2,649	(80)	40.43	32.58	9.82	17.18	0.0011
Non-Hispanic Black			465	(14)	460	(14)	36.74	26.09	13.26	23.91	
Non-Hispanic Other			56	(2)	56	(2)	32.14	33.93	8.93	25.00	
Hispanic			114	(3)	113	(3)	41.59	23.01	8.85	26.55	
Unknown			22	(1)	22	(1)	22.73	40.91	13.64	22.73	
Marital status											
Married			2,508	(75)	2,489	(75)	43.51	31.90	9.04	15.55	<0.0001
Single			232	(7)	229	(7)	34.06	27.07	10.04	28.82	
Separated/divorced			174	(5)	173	(5)	39.88	26.01	10.40	23.70	
Widowed			234	(7)	230	(7)	16.96	31.74	19.13	32.17	
Unknown			180	(5)	179	(5)	22.91	35.20	16.20	25.70	
Insurance											
None			26	(1)	26	(1)	42.31	26.92	11.54	19.23	<0.0001
Private			2,384	(72)	2,365	(72)	42.79	31.63	9.56	16.03	
Public			570	(17)	564	(17)	31.56	28.90	13.12	26.42	
Other			94	(3)	94	(3)	53.19	24.47	7.45	14.89	
Unknown			254	(8)	251	(8)	23.51	38.25	11.55	26.69	
Education^4^											
Educated			2,431	(73)	2,409	(73)	41.22	31.51	9.46	17.81	0.0062
Not educated			880	(26)	874	(26)	35.81	30.78	12.59	20.82	
Poverty^4^											
Not in poverty			2,546	(77)	2,529	(77)	40.45	31.99	9.65	17.91	0.0275
In poverty			765	(23)	754	(23)	37.53	29.05	12.47	20.95	
Working class status^4^											
Non-working class			1,738	(52)	1,724	(52)	40.14	33.29	9.40	17.17	0.0165
Working class			1,573	(47)	1,559	(47)	39.38	29.12	11.29	20.21	
Urban-rural residence^4^											
100% urban			2,196	(66)	2,174	(66)	39.93	31.05	9.84	19.18	0.4804
Urban-rural mix			853	(26)	848	(26)	40.57	31.13	10.85	17.45	
100% rural			262	(8)	261	(8)	36.02	34.10	12.26	17.62	
Registry											
California			507	(15)	503	(15)	43.74	25.25	12.52	18.49	<0.0001
Colorado			509	(15)	507	(15)	44.18	28.60	10.45	16.77	
Illinois			522	(16)	521	(16)	36.28	31.48	10.36	21.88	
Louisiana			530	(16)	529	(16)	42.53	26.47	11.53	19.47	
New York			445	(13)	435	(13)	34.94	40.69	9.43	14.94	
Rhode Island			464	(14)	459	(14)	36.17	35.29	8.71	19.83	
South Carolina			351	(11)	346	(10)	38.73	35.26	7.80	18.21	

1: Sample size for analysis of conservative versus definite therapy.

2: Sample size for dividing conservative treatment into hormone therapy and watchful waiting (excludes 28 observations missing dates for hormone therapy). Used for calculating proportions in this table.

3: Probability from a two-sided X^2 ^test comparing the distribution of treatments across levels of the variable.

4: Information missing for 17 individuals.

Except for urban-rural residence, all other variables considered in the bivariate analysis were found to be statistically significantly associated with mode of initial treatment (Tables 1 and 2). The proportion of prostate cancers treated surgically decreased dramatically with age. As the proportion of radical prostatectomy decreased, the proportion of radiation therapy increased until age 75-79. Thereafter, conservative treatment predominated. Radical prostatectomy was the predominant mode of initial treatment for all race/ethnic groups except non-Hispanic other. Non-Hispanic white men were least likely to receive conservative management. The proportions of both types of definitive treatment were highest among married men. The overwhelming majority of men in our sample had private insurance. Men with public insurance were least likely to receive surgical treatment and most likely to receive conservative management. Patients residing in high education census tracts were more apt to have been surgically treated, as were patients residing in non-poverty census tracts and patients in non-working class census tracts. Treatment patterns varied geographically. The proportion of men with clinically localized prostate cancer who underwent radical prostatectomy was highest in Colorado (44.2%) and lowest in New York (34.9%). Receipt of radiation therapy was highest in New York (40.7%) and lowest in California (25.3%). Less absolute variability was observed in the proportion of men who underwent conservative management (range: 26.1% in New York to 32.4% in Illinois). The predominant mode of radiotherapy was external beam. Of the 1,037 patients treated with radiation, 74.8% received external beam radiation, 16.7% received brachytherapy, virtually all of which was permanent implant, and 8.5% received both (data not shown).

**Table 2:** Distribution of initial treatment by clinical and tumor characteristics.

					Percent receiving each therapy				
						
Characteristic	**Number**^**1**^	(%)	**Number**^**2**^	(%)	Radical prostatectomy	Radiation therapy	Hormonal therapy	Watchful waiting	**P**^**3**^
Screen-detected									
Yes	2,106	(63)	2,092	(63)	43.45	34.46	9.70	12.38	<0.0001
No	864	(26)	855	(26)	31.70	25.73	10.76	31.81	
Unknown	358	(11)	353	(11)	36.83	27.20	12.46	23.51	
DRE results									
Normal	1,312	(39)	1,301	(39)	42.51	28.67	9.76	19.06	<0.0001
Abnormal	1,086	(33)	1,081	(33)	37.47	38.48	11.84	12.21	
Equivocal/unk	930	(28)	918	(28)	38.34	27.02	9.15	25.49	
PSA value									
0-4	290	(9)	289	(9)	43.94	28.72	5.19	22.15	<0.0001
>4-10	1,603	(48)	1,599	(48)	46.78	33.27	7.57	12.38	
>10-20	617	(19)	608	(18)	33.22	38.82	12.01	15.95	
>20-50	260	(8)	254	(8)	20.87	35.04	22.83	21.26	
50+	111	(3)	111	(3)	20.72	27.03	24.32	27.93	
Unknown	447	(13)	439	(13)	35.76	15.26	10.25	38.72	
Gleason score									
2-4	352	(11)	347	(11)	31.70	23.92	7.49	36.89	<0.0001
5-7	2,475	(74)	2,459	(75)	44.57	30.87	8.95	15.62	
8-10	289	(9)	285	(9)	25.96	40.70	21.40	11.93	
Unknown	212	(6)	209	(6)	14.35	37.80	15.31	32.54	
Comorbidity score									
0	2,772	(83)	2,749	(83)	41.36	32.34	9.28	17.02	<0.0001
1	441	(13)	437	(13)	33.87	27.69	11.90	26.54	
2+	115	(3)	114	(3)	21.93	23.68	28.07	26.32	

1: Sample size for analysis of conservative versus definite therapy.

2: Sample size for dividing conservative treatment into hormone therapy and watchful waiting (excludes 28 observations missing dates for hormone therapy). Used for calculating proportions in this table.

3: Probability from a two-sided X^2 ^test comparing the distribution of treatments across levels of the variable.

The distribution of initial treatment by clinical and tumor characteristics is shown in Table 2. In general, less aggressive tumors, that is, tumors that were screen detected or were not palpable by DRE or had low PSA values or low Gleason scores, were more likely to be treated surgically than with radiation therapy. Both types of definitive treatment decreased as the comorbidity score increased.

Crude and adjusted odds ratios for receipt of conservative management are shown in Table 3. No statistically significant differences in receipt of conservative management were observed for census-based socioeconomic indicators after adjustment for other variables. As expected, the likelihood of receiving conservative management increased dramatically with age. The odds of receiving conservative management among patients 60-64 years of age were 65% greater than among men below age 60. The corresponding odds for patients in their 80 s were 27 times greater than for patients below age 60. Black non-Hispanics were more likely to receive conservative therapy than White non-Hispanics. Unmarried patients were more likely to receive conservative therapy than those that were married. Patients with public insurance were more likely to receive conservative treatment than patients with private insurance. Men with localized prostate cancer residing in Louisiana and South Carolina were less likely to receive conservative management than men residing in California. Differences in the likelihood of conservative management did not differ significantly between California and the states of Illinois, New York, and Rhode Island, whereas the likelihood was marginally higher in Colorado. Men with screen-detected prostate cancer were less likely to receive conservative therapy than men whose cancer was not screen-detected. Patients whose DRE results were abnormal were less likely to receive conservative therapy than patients with normal DRE results. PSA value was associated with receipt of conservative treatment but only at very high values, that is, at values above 20. Patients whose tumors were moderately or poorly differentiated (Gleason score 5-10) were less likely to receive conservative treatment than patients with well differentiated tumors (Gleason score 2-4). The likelihood of receiving conservative treatment increased with the number of comorbidities.

**Table 3:** Percentage (%) distributions and odds ratios for receipt of conservative therapy (n = 3,328).

Characteristic	%	**Crude **^**1**^	OR (95% CI)	**Adj.**^**2**^	OR (95% CI)
Age at diagnosis					
<60	12.13	1.00	(referent)	1.00	(referent)
60-64	17.96	1.59	(1.15 - 2.20)	1.65	(1.18 - 2.33)
65-69	20.38	1.85	(1.37 - 2.52)	1.90	(1.38 - 2.63)
70-74	32.58	3.50	(2.63 - 4.71)	3.35	(2.46 - 4.59)
75-79	46.71	6.35	(4.70 - 8.67)	6.76	(4.87 - 9.47)
>= 80	82.49	34.13	(23.00 - 51.68)	27.09	(17.66 - 42.31)
Race/ethnicity					
Non-Hispanic White	27.59	1.00	(referent)	1.00	(referent)
Non-Hispanic Black	37.85	1.60	(1.30 - 1.96)	1.65	(1.28 - 2.13)
Non-Hispanic Other	33.93	1.35	(0.76 - 2.33)	1.28	(0.66 - 2.40)
Hispanic	35.96	1.47	(0.99 - 2.17)	1.56	(0.98 - 2.45)
Unknown	36.36	1.50	(0.60 - 3.52)	1.02	(0.31 - 3.02)
Marital status					
Married	25.16	1.00	(referent)	1.00	(referent)
Not Married	42.81	2.23	(1.86 - 2.67)	1.72	(1.39 - 2.14)
Unknown	42.22	2.17	(1.59 - 2.96)	1.70	(1.15 - 2.49)
Insurance					
Private/other	26.03	1.00	(referent)	1.00	(referent)
None	30.77	1.26	(0.52 - 2.83)	2.31	(0.89 - 5.52)
Public	40.18	1.91	(1.58 - 2.31)	1.51	(1.20 - 1.89)
Unknown	38.98	1.82	(1.39 - 2.37)	1.60	(1.14 - 2.25)
Registry					
California	31.56	1.00	(referent)	1.00	(referent)
Colorado	27.50	0.82	(0.63 - 1.08)	1.37	(0.99 - 1.90)
Illinois	32.38	1.04	(0.80 - 1.35)	0.95	(0.68 - 1.32)
Louisiana	31.13	0.98	(0.75 - 1.28)	0.59	(0.42 - 0.82)
New York	26.07	0.77	(0.58 - 1.01)	0.85	(0.60 - 1.20)
Rhode Island	29.31	0.90	(0.68 - 1.18)	1.12	(0.80 - 1.56)
South Carolina	27.07	0.81	(0.60 - 1.09)	0.63	(0.43 - 0.91)
Screen-detected					
Yes	22.60	1.00	(referent)	1.00	(referent)
No	43.17	2.60	(2.20 - 3.08)	2.32	(1.86 - 2.91)
Unknown	36.87	2.00	(1.57 - 2.53)	1.47	(1.08 - 1.99)
DRE results					
Normal	29.42	1.00	(referent)	1.00	(referent)
Abnormal	24.40	0.77	(0.65 - 0.93)	0.67	(0.54 - 0.84)
Equivocal/unknown	35.48	1.32	(1.10 - 1.58)	0.91	(0.73 - 1.15)
PSA value					
0-4	27.59	1.00	(referent)	1.00	(referent)
>4-10	20.15	0.66	(0.50 - 0.88)	0.69	(0.50 - 0.97)
>10-20	29.01	1.07	(0.79 - 1.47)	0.80	(0.56 - 1.15)
>20-50	45.38	2.18	(1.53 - 3.12)	1.41	(0.93 - 2.15)
50+	52.25	2.87	(1.83 - 4.53)	1.73	(1.02 - 2.94)
Unknown	49.89	2.61	(1.91 - 3.60)	1.64	(1.12 - 2.41)
Gleason score					
2-4	45.17	1.00	(referent)	1.00	(referent)
5-7	25.05	0.41	(0.32 - 0.51)	0.45	(0.34 - 0.59)
8-10	34.26	0.63	(0.46 - 0.87)	0.49	(0.33 - 0.72)
Unknown	48.58	1.15	(0.88 - 1.61)	0.75	(0.50 - 1.13)
Comorbidity score					
0	26.91	1.00	(referent)	1.00	(referent)
1	39.00	1.74	(1.41 - 2.14)	1.44	(1.12 - 1.86)
2+	54.78	3.29	(2.26 - 4.81)	2.59	(1.67 - 4.03)

1: OR = odds ratio; CI = confidence interval.

2: Adjusted for factors shown in the table.

Crude and adjusted odds ratios for receipt of radiation therapy among patients receiving definitive treatment are shown in Table 4. After adjustment for the other variables in the model, no statistically significant differences were observed for census- based socioeconomic indicators, type of insurance, whether the cancer was screen detected, or comorbidity score. Race/ethnicity also appeared to be a non-significant factor. However, two alternative models fit the data equally well. The first model contains an age-race/ethnicity interaction. The second model does not contain race/ethnicity. The odds ratios for all other factors vary negligibly between the two models.

**Table 4:** Percentage (%) distributions and odds ratios for receipt of radiation therapy among patients receiving definitive treatment (n = 2,347).

Characteristic	%	**Crude **^**1**^	OR (95% CI)	**Adj.**^**2**^	OR (95% CI)	**Adj.**^**3**^	OR (95% CI)
				**Model 1**		**Model 2**	
Age-Race/ethnicity							
Interaction^4^							
<60, Non-Black		1.00	(referent)	1.00	(referent)		
<60, NH Black		2.98	(1.77 - 4.96)	2.62	(1.50 - 4.52)		
60-64, Non-Black		2.01	(1.42 - 2.88)	1.95	(1.36 - 2.83)		
60-64, NH Black		2.80	(1.52 - 5.03)	2.53	(1.32 - 4.72)		
65-74, Non-Black		7.20	(5.38 - 9.79)	7.55	(5.57 - 10.40)		
65-74, NH Black		5.14	(3.24 - 8.18)	5.19	(3.15 - 8.59)		
>= 75, Non-Black		68.08	(41.57 - 116.45)	69.73	(41.94 - 120.93)		
>= 75, NH Black		41.09	(13.58 - 178.13)	43.31	(13.28 - 196.39)		
Age at diagnosis							
<60	17.20	1.00	(referent)			1.00	(referent)
60-64	25.54	1.65	(1.22 - 2.25)			1.63	(1.18 - 2.26)
65-69	39.76	3.18	(2.41 - 4.22)			3.37	(2.52 - 4.54)
70-74	69.25	10.84	(8.08 - 14.66)			12.22	(8.94 - 16.86)
>= 75	91.32	50.63	(32.27 - 82.56)			54.47	(34.11 - 90.27)
Marital status							
Married	42.30	1.00	(referent)	1.00	(referent)	1.00	(referent)
Not Married	49.18	1.32	(1.05 - 1.65)	1.44	(1.10 - 1.88)	1.51	(1.15 - 1.98)
Unknown	60.58	2.10	(1.41 - 3.16)	2.20	(1.36 - 3.58)	2.22	(1.36 - 3.65)
Registry							
California	36.60	1.00	(referent)	1.00	(referent)	1.00	(referent)
Colorado	39.30	1.12	(0.83 - 1.52)	1.57	(1.10 - 2.25)	1.60	(1.11 - 2.31)
Illinois	46.46	1.50	(1.11 - 2.04)	1.57	(1.09 - 2.27)	1.71	(1.17 - 2.49)
Louisiana	38.36	1.08	(0.80 - 1.46)	1.06	(0.73 - 1.53)	1.07	(0.74 - 1.56)
New York	53.80	2.02	(1.49 - 2.75)	2.40	(1.66 - 3.49)	2.78	(1.91 - 4.06)
Rhode Island	49.39	1.69	(1.24 - 2.30)	2.00	(1.39 - 2.90)	1.98	(1.36 - 2.89)
South Carolina	47.66	1.58	(1.14 - 2.19)	2.12	(1.43 - 3.14)	2.35	(1.58 - 3.51)
DRE results							
Normal	40.28	1.00	(referent)	1.00	(referent)	1.00	(referent)
Abnormal	50.67	1.52	(1.26 - 1.84)	1.19	(0.95 - 1.50)	1.17	(0.93 - 1.48)
Equivocal/unknown	41.33	1.05	(0.85 - 1.29)	0.78	(0.60 - 1.02)	0.77	(0.59 - 1.00)
PSA value							
0-4	39.52	1.00	(referent)	1.00	(referent)	1.00	(referent)
>4-10	41.56	1.09	(0.81 - 1.47)	1.01	(0.71 - 1.44)	1.06	(0.74 - 1.53)
>10-20	53.88	1.79	(1.28 - 2.50)	1.57	(1.06 - 2.34)	1.64	(1.09 - 2.46)
20+	61.00	2.40	(1.61 - 3.58)	2.14	(1.34 - 3.45)	2.17	(1.35 - 3.52)
Unknown	29.91	0.65	(0.44 - 0.97)	0.45	(0.27 - 0.73)	0.46	(0.28 - 0.75)
Gleason score							
2-4	43.01	1.00	(referent)	1.00	(referent)	1.00	(referent)
5-7	40.92	0.92	(0.68 - 1.24)	0.76	(0.54 - 1.09)	0.76	(0.53 - 1.09)
8-10	61.05	2.08	(1.39 - 3.13)	1.53	(0.94 - 2.48)	1.59	(0.97 - 2.62)
Unknown	72.48	3.49	(2.12 - 5.86)	5.59	(3.09 - 10.34)	5.64	(3.08 - 10.52)

1: OR = odds ratio; CI = confidence interval.

2,3: Adjusted for factors shown in the table.

4: NH = Non-Hispanic, non-Black includes all race/ethnic groups other than NH Blacks.

Within the interaction term, race was dichotomized into non-Hispanic Black and all others. Within both race/ethnicity categories, the likelihood of receiving radiation therapy as opposed to radical prostatectomy increased with age although much less steeply for non-Hispanic Blacks (Table 5). However, the odds of receiving radiation therapy for non-Hispanic Blacks under age 60 were 2.6 times greater than the odds for other men of the same age. At ages 60 and greater, the odds of receiving radiation therapy did not vary by race/ethnicity.

**Table 5:** Alternate presentations of odds ratios by age and race for receipt of radiation therapy among patients receiving definitive treatment (model 1 from Table 4).

	Non-Black		Non-Hispanic Black	
Age	OR	(95% CI)	OR	(95% CI)
OR(Age) within Strata of Race				
<60	1.00	referent	1.00	referent
60-64	1.95	(1.36 - 2.83)	0.97	(0.46 - 2.00)
65-74	7.55	(5.57 - 10.40)	1.98	(1.08 - 3.70)
75+	69.73	(41.94 - 120.93)	16.53	(4.80 - 77.66)
				
OR(Race) within Strata of Age				
Age				
<60	1.00	referent	2.62	(1.50 - 4.52)
60-64	1.00	referent	1.30	(0.69 - 2.37)
65-74	1.00	referent	0.69	(0.44 - 1.06)
75+	1.00	referent	0.62	(0.18 - 2.90)

Among men who received definitive treatment, unmarried patients were more likely to receive radiation therapy than married patients. With the exception of Louisiana, patients residing in the remaining five states were significantly more likely to receive radiation therapy than patients residing in California. Prostate cancer patients with PSA values above 10 were more likely to receive radiation therapy. Men with poorly differentiated tumors (Gleason score 8-10) were more likely to receive radiation therapy than men with well differentiated tumors (Gleason score 2-4).

The survival analysis was limited to 3,297 patients. Three patients for whom information on year of last contact was missing were excluded from the 3,300 patients with known treatment modality (n = 3,300; see Table 1). All sociodemographic, economic, and clinical characteristics shown in Tables 1 and 2 were found to be significantly associated with survival, as was treatment modality. Five-year survival rates and crude and adjusted hazard ratios for factors retained in the final model are shown in Table 6. The proportional hazards assumption for comorbidity was found to hold for the following intervals: ≤ 1 year post-diagnosis; >1 to 3 years post-diagnosis; and >3 years post-diagnosis.

**Table 6:** Five-year survival and crude and adjusted hazard ratios by sociodemograhic and clinical factors (n = 3,297).

Characteristic			**5 yr Survival**^**1**^**(%)**	**Crude Hazard Ratio (95% CI)**^**2**^		**Adjusted**^**3 **^**Hazard Ratio (95% CI)**	
Age at diagnosis							
<60			95.06	1.00	(referent)	1.00	(referent)
60-64			93.38	1.38	(0.86 - 2.20)	1.28	(0.80 - 2.04)
65-69			87.92	2.59	(1.73 - 3.89)	2.10	(1.39 - 3.18)
70-74			85.60	3.33	(2.24 - 4.95)	2.28	(1.51 - 3.46)
75-79			74.67	6.18	(4.18 - 9.14)	3.67	(2.41 - 5.60)
>= 80			50.82	13.00	(8.78 - 19.25)	5.29	(3.43 - 8.16)
Race/ethnicity							
Non-Hispanic White			85.42	1.00	(referent)	1.00	(referent)
Non-Hispanic Black			78.78	1.47	(1.18 - 1.83)	1.17	(0.92 - 1.48)
Non-Hispanic Other			94.51	0.31	(0.10 - 0.95)	0.35	(0.11 - 1.10)
Hispanic			92.65	0.51	(0.27 - 0.96)	0.53	(0.28 - 1.00)
Unknown			78.25	1.40	(0.52 - 3.74)	1.29	(0.46 - 3.61)
Marital status							
Married			86.36	1.00	(referent)	1.00	(referent)
Not Married			78.58	1.69	(1.40 - 2.05)	1.21	(1.00 - 1.48)
Unknown			86.39	0.90	(0.59 - 1.35)	0.67	(0.43 - 1.03)
Registry							
California			86.03	1.00	(referent)	1.00	(referent)
Colorado			89.97	0.79	(0.56 - 1.10)	0.94	(0.66 - 1.33)
Illinois			82.36	1.35	(1.00 - 1.82)	1.01	(0.74 - 1.38)
Louisiana			78.80	1.69	(1.28 - 2.25)	1.37	(1.02 - 1.84)
New York			86.90	1.01	(0.73 - 1.40)	1.10	(0.78 - 1.54)
Rhode Island			88.38	0.91	(0.65 - 1.27)	0.82	(0.58 - 1.16)
South Carolina			79.79	1.72	(1.21 - 2.44)	1.54	(1.06 - 2.24)
PSA value							
0-4			90.49	1.00	(referent)	1.00	(referent)
>4-10			89.28	1.00	(0.69 - 1.44)	1.07	(0.73 - 1.55)
>10-20			84.65	1.50	(1.01 - 2.21)	1.20	(0.81 - 1.79)
20+			75.77	2.43	(1.64 - 3.60)	1.38	(0.92 - 2.09)
Unknown			73.16	2.65	(1.80 - 3.90)	1.82	(1.22 - 2.70)
Gleason score							
2-4			83.90	1.00	(referent)	1.00	(referent)
5-7			86.73	1.10	(0.81 - 1.50)	1.35	(0.98 - 1.85)
8-10			73.69	2.21	(1.54 - 3.19)	1.96	(1.34 - 2.86)
Unknown			74.52	2.29	(1.55 - 3.38)	1.69	(1.13 - 2.51)
Treatment							
Radical prostatectomy			93.68	1.00	(referent)	1.00	(referent)
Radiation therapy			85.99	2.55	(1.97 - 3.31)	1.66	(1.24 - 2.21)
Hormonal therapy			65.24	6.75	(5.13 - 8.88)	2.83	(2.06 - 3.90)
Watchful waiting			75.47	4.73	(3.65 - 6.14)	2.30	(1.70 - 3.12)
Comorbidity score^4^							
≤1 year post diagnosis							
0			86.69^#^	1.00	(referent)	1.00	(referent)
1			79.68^#^	2.84	(1.39 - 5.81)	2.16	(1.05 - 4.41)
2+			62.62^#^	9.90	(4.78 - 20.88)	6.39	(3.04 - 13.42)
>1 to 3 years post diagnosis							
0				1.00	(referent)	1.00	(referent)
1				1.56	(1.08 - 2.26)	1.20	(0.83 - 1.74)
2+				3.99	(2.52 - 6.31)	2.67	(1.68 - 4.25)
>3 years post diagnosis							
0				1.00	(referent)	1.00	(referent)
1				1.48	(1.09 - 2.00)	1.22	(0.90 - 1.66)
2+				2.16	(1.30 - 3.58)	1.47	(0.88 - 2.45)

1: 5-year survival following diagnosis conditional on having survived the first six months following diagnosis.

2: OR = odds ratio; CI = confidence interval.

3: Adjusted for factors shown in the table.

4: Hazard associated with comorbidity is time dependent.

#: 5 year survival percent (not stratified by time since diagnosis)

Risk of death from any cause increased substantially with age. Hispanic men had a significantly lower risk of death than non-Hispanic white men; this difference remained borderline significant after adjusting for other factors (p = 0.0511). Married men had a lower risk of death than unmarried men; this difference also remained borderline significant after adjustment (p = 0.0551). Five-year survival rates varied by state of residence, from 78.8% for Louisiana to 90.0% for Colorado. After adjusting for other factors, the hazard ratio remained significantly higher (significantly lower survival) for Louisiana and South Carolina. Risk of death increased with increasing PSA levels. However, the increase in risk attenuated after adjustment for other factors; only men with unknown PSA levels remained at significantly increased risk. Men with undifferentiated tumors, Gleason score 8-10, or with tumors of unknown grade were at significantly higher risk of death even after adjusting for other factors. Five-year survival varied significantly with treatment modality; survival was highest for patients who received radical prostatectomy (93.7%) and lowest for patients who received hormone therapy (65.2%). For patients who received radiation therapy and for patients who underwent watchful waiting, the five-year survival rates were 86.0% and 74.5%, respectively. Even after adjusting for factors that were predictive of treatment, patients who did not receive a radical prostatectomy were significantly more likely to die. The risk of death associated with comorbid conditions attenuated with time since diagnosis; the attenuation was greater after adjusting for other factors. Within the first year following diagnosis, the hazard ratio was 2.16 for patients with a comorbidity score of 1 and 6.39 for patients with a score of 2 or more. One to three years post diagnosis, patients with a comorbidity score of 1 were no longer at increased risk of dying compared to patients with no comorbidity, whereas patients with a comorbidity score of 2 or more were still at significantly increased risk but the hazard ratio was reduced to 2.67. Three years after diagnosis, comorbidity at diagnosis was no longer associated with survival. After adjustment for other factors, no statistically significant differences in survival were observed for insurance, education, poverty, working class status, urban-rural residence, whether the tumor was screen detected, or digital rectal exam (DRE) results.

We found further survival differences within the group receiving radiation therapy. Compared to radical prostatectomy, the adjusted hazard ratio and 95% confidence intervals for brachytherapy, external beam radiation, and combined radiotherapy were 0.58 (0.29, 1.16), 1.90 (1.41, 2.55), and 1.70 (0.93, 3.08), respectively. Thus the apparent survival advantage of radical prostatectomy compared to radiation therapy was limited to external beam radiation. The adjusted hazard ratios for the covariates shown in Table 6 did not change appreciably with the introduction of the additional treatment terms into the model (data not shown).

## Discussion

The rates of radical prostatectomy and radiation therapy observed in our study are comparable to those observed for patients in areas covered by the National Cancer Institute's (NCI) Surveillance, Epidemiology and End Results (SEER) program diagnosed in 1997 with a first primary, localized prostate cancer; that is 35.5% and 30.5% for radical prostatectomy and radiation therapy, respectively [12]. However, these rates differ from what was observed in the NCI Prostate Cancer Outcomes Study [11], which showed higher rates of radical prostatectomy and lower rates of radiation therapy. Part of the difference may reflect temporal trends, since the NCI study was based on 1994 and 1995 diagnosed cases; the study also did not include all SEER areas. Among 86,298 men with stage I and II prostate cancers reported to the National Cancer Data Base (NCDB) with 1998 diagnoses, 39% were treated with surgery, 42% received radiation, 18% were managed conservatively (watchful waiting with or without hormone therapy), and 1% received other specified therapy. Of the patients treated surgically, 78% received a radical prostatectomy, or 31% overall [13]. Whereas we observed a higher radical prostatectomy rate than seen in NCDB, our radiation therapy rate was considerably lower. In addition to changing temporal trends, this may also reflect differences between population-based data and data based on selected hospitals (i.e., American College of Surgeons (ACoS) approved hospitals), albeit covering all states. Patients treated at ACoS approved hospitals may have better insurance and better socioeconomic status in general [14].

Geographic difference in treatment patterns persisted by state after adjustment for other factors, both in terms of definitive treatment versus conservative treatment and radiation therapy versus radical prostatectomy. New York was the only state in which radiation therapy was the most common treatment modality. State differences have been reported in a number of studies [11,15,16], including generally higher prostatectomy rates in the Mountain-Pacific region than in the Northeast and the converse for radiation therapy [17,18]. Interstate differences in treatment patterns probably reflect local practice patterns [19] as well as access to treatment facilities.

Our observation that black patients were more likely to be treated conservatively than white patients is generally consistent with previous studies [11,14,16,17,20-23] and has been related to the lower likelihood of black men to undergo radical prostatectomy [11,16,17,20,22-25]. We found that among men receiving definitive therapy, treatment selection did not differ by race except in patients under age 60. In this age group black men were much more likely to receive radiation therapy than radical prostatectomy. Race-related differences in prostate cancer treatment patterns remain poorly understood. If social and economic disparities were the underlying factors determining the racial variation, then one would also expect lower utilization of radiation therapy and hormone treatment, which has not been consistently observed.

Consistent with treatment guidelines [26], we found that clinical factors such as life expectancy, PSA level, Gleason score, and competing medical conditions affect prostate cancer treatment selection. We found that unmarried men were more likely to receive conservative treatment, which was also observed by Harlan et al (2001). However, in their study, marital status was not associated with treatment selection among men receiving definitive treatment, whereas, in ours, unmarried men were more likely to receive radiation therapy. We also found that men with public health insurance were more likely to receive conservative treatment, which was not observed by Harlan et al (2001). Among men receiving definitive treatment, no association was observed with type of insurance. After multivariable adjustment, we found no association between treatment and area-based socioeconomic measures.

Survival of patients in this study was related to age, marital status, Gleason score, comorbid illness, type of treatment, and state of residence; unlike in some other studies, survival did not differ by race [27-29]. Although patients in this study were diagnosed with clinically localized disease, 9% had a Gleason score of 8 to 10, which is very predictive of nodal involvement [30] and, thus, lower survival. The observed attenuation in the effect of comorbidity on all-cause mortality with time may reflect under-ascertainment of comorbidities in our study since a strong association with comorbidity score and ten-year all-cause mortality has been previously reported [31].

To date, only three completed randomized trials have compared effectiveness between major prostate cancer treatment categories; none of these enrolled men with primarily PSA-detected cancer, which limits their applicability to current practice [32]. Two trials compared radical prostatectomy with watchful waiting. In the Veterans Administration Cooperative Urological Research Group (VACURG) study [33], the median overall survival was 10.6 years for the radical prostatectomy group and 8 years for the watchful waiting group after a median follow-up of 23 years. Results were not statistically significant, but this study had limited power since it only included 142 patients. The Scandinavian trial, which included 695 men, did not find a statistically significant difference in overall survival at 5 or 8 years of follow-up [34]; but after 10 years found a 5% absolute risk reduction (0.74 relative risk, p = 0.04) in overall mortality in the radical prostatectomy group [35]. At 12 years, the all-cause mortality relative risk was attenuated and no longer statistically significant [36]. In the same trial, radical prostatectomy reduced prostate cancer-specific mortality as well as risk of distant metastases; these benefits were evident at 5 years of follow-up and showed little or no further increase in benefit 10 or more years after surgery [34-36]. The third trial, which compared radical prostatectomy with external beam radiation therapy, did not evaluate survival but found that surgery was more effective in preventing progression, recurrence, or distant metastases in patients with non-PSA detected cancers [32].

In our study five-year overall survival for radical prostatectomy was significantly higher than for the other three treatment modalities. However, when subdividing radiation therapy, we found that survival following brachytherapy was comparable. Although there appeared to be a slight survival advantage for radiation therapy compared to conservative management, even when limited to external beam radiation, and for watchful waiting compared to hormone therapy, these differences were not statistically significant after adjusting for other factors. Our findings are generally consistent with those from other observational studies, which have shown an overall survival advantage for definitive treatment versus watchful waiting [37] and no overall survival advantage for hormone therapy compared to watchful waiting [38]. A recent trial of immediate versus deferred androgen deprivation, which our study did not examine, found a modest increase in overall survival for immediate androgen deprivation [39]. In observational studies such as ours, caution must be exercised in attributing survival differences to treatment received since healthier patients are more likely to be treated aggressively and residual confounding cannot be discounted [40,41].

Our study was subject to several limitations. Because of our reliance on medical records, we were not able to distinguish between actual watchful waiting and surveillance, the latter being an active management process where "curative" treatment may be applied if progression is noted. This may explain why we observed somewhat better survival for watchful waiting than for hormone therapy.

Significant advances have been made in the treatment of prostate cancer since 1997. Radical prostatectomy is increasingly being performed laparoscopically. Three dimensional conformal radiotherapy (3D-CRT), which had been the accepted standard of external beam therapy until the past 5-7 years, is being supplanted by intensity modulated radiation therapy and stereotactic body radiotherapy, and to a lesser extent, proton beam radiotherapy. However, although practice patterns may have changed, understanding the factors associated with choice of definite treatment versus conservative management remains relevant.

Another limitation is lack of information on patient preferences, their attitudes toward the medical system as well as on the patient and physician decision-making process. Prostate cancer treatment selection is influenced by external recommendations, concerns about effects of treatment on quality of life, characteristics of the treatment itself, economic and logistic considerations, as well as by personal perceptions and values [42,43]. In one study prostate cancer patients reported that the physician was the most important factor influencing their treatment decision [44]. Other studies found that treatment selection for prostate cancer was related to the treatment options that were discussed [11], the information patients received [45], and the extent to which patients were involved in the decision-making process [46]. Patient involvement in the choice of treatment is particularly important in early stage prostate cancer because the various treatment regimens often involve significant side effects [47-49] that impact quality of life and patient satisfaction with treatment outcomes [50-52].

Although this study included samples from seven states and resulted in a fairly large sample size, results may not be generalizable to the entire country. Additionally, the study did not over-sample minorities. Therefore, the number of non-white and Hispanic prostate cancer patients was still relatively small and may not have allowed us to adequately assess racial and ethnic differences. Furthermore, the five-year lag between cancer diagnosis and data collection resulted in difficulty retrieving some medical information. Reliance on face sheets in medical records for comorbidity information probably led to under-ascertainment of comorbidities. The study also did not have access to individual-level measures of socioeconomic status and relied instead on area-derived Census measures. This may have resulted in inadequate adjustment for socioeconomic effects. Finally, we were unable to examine cause-specific mortality in addition to overall mortality.

The CDC-NPCR Patterns of Care study follows the Institute of Medicine recommendations that data systems such as SEER and NPCR be used to conduct surveillance of treatment in the United States [7]. Although a number of prostate cancer patterns of care studies have been conducted using data from the SEER program, this is the first such study using data from the NPCR. There is some evidence to suggest that patients residing in SEER areas are somewhat more affluent [53] and may have greater access to high quality cancer care. Of the seven states that participated in this study, none was part of the SEER program in 1997, and several had not participated in previous patterns of care studies (e.g., New York). For these states, this study was very significant in that it provided them with valuable baseline treatment information and enhanced their capacity to conduct treatment surveillance. The present study also serves as the foundation for future NPCR patterns of care studies, one of which is currently in progress [54]. The second NPCR Patterns of Care study addresses several limitations of the current study. In particular, sampling was stratified by race, and more detailed treatment data are being collected, including radiation dose.

## Conclusions

The fact that geographic variation remained even after adjusting for other factors, both in treatment patterns as well as in survival, suggests true regional differences in how physicians and patients select treatment options. Factors affecting treatment choice and treatment effectiveness need to be further explored in future population-based studies. Prostate cancer treatment contributes significantly to national healthcare costs because of the sheer number of men who are affected [55], with the cost per patient dependent on the type of treatment received [56]. Given the current healthcare reform discussions, a thorough understanding of the factors driving treatment selection is both critical and timely [57].

## Competing interests

The authors declare that they have no competing interests. This study was funded in large part through cooperative agreements awarded by the Division of Cancer Prevention and Control, Centers for Disease Control and Prevention to the states of California, Colorado, Illinois, Louisiana, New York (U58/CCU220322), Rhode Island, and South Carolina. The findings and conclusions in this manuscript are those of the authors and do not necessarily represent the official position of the Centers for Disease Control and Prevention.

## Authors' contributions

MJS, ARK, JLF, JPF and TS contributed to the development of the PoC1 study protocol. RRG was responsible for study coordination among registries. MJS and ARK were responsible for the data analyses that are presented in this manuscript. MJS was responsible for the drafting of the manuscript; RRG and MCH contributed significantly to the literature review; and all authors contributed to the final manuscript. All authors read and approved the final manuscript.

## Pre-publication history

The pre-publication history for this paper can be accessed here:

http://www.biomedcentral.com/1471-2407/10/152/prepub
